# The Management of Patients With Idiopathic Pulmonary Fibrosis

**DOI:** 10.3389/fmed.2018.00148

**Published:** 2018-07-02

**Authors:** Paolo Spagnolo, Argyris Tzouvelekis, Francesco Bonella

**Affiliations:** ^1^Respiratory Disease Unit, Department of Cardiac, Thoracic and Vascular Sciences, University of Padova, Padova, Italy; ^2^Division of Immunology, Biomedical Sciences Research Center “Alexander Fleming”, Athens, Greece; ^3^Interstitial and Rare Lung Disease Unit, Ruhrlandklinik, University of Duisburg-Essen, Essen, Germany

**Keywords:** idiopathic pulmonary fibrosis, pharmacologic treatment, pirfenidone, nintedanib, non-pharmacological treatment, therapy

## Abstract

Idiopathic pulmonary fibrosis (IPF), the most common form of fibrosing idiopathic interstitial pneumonia, is an inexorably progressive disease with a 5-year survival of ~20%. In the last decade, our understanding of disease pathobiology has increased significantly and this has inevitably impacted on the approach to treatment. Indeed, the paradigm shift from a chronic inflammatory disorder to a primarily fibrotic one coupled with a more precise disease definition and redefined diagnostic criteria have resulted in a massive increase in the number of clinical trials evaluating novel candidate drugs. Most of these trials, however, have been negative, probably because of the multitude and redundancy of cell types, growth factors and profibrotic pathways involved in disease pathogenesis. As a consequence, until recently IPF has lacked effective therapies. Finally, in 2014, two large phase 3 clinical trials have provided robust evidence that pirfenidone, a compound with anti-fibrotic, anti-oxidant and anti-inflammatory properties, and nintedanib, a tyrosine kinase inhibitor with selectivity for vascular endothelial growth factor, platelet-derived growth factor and fibroblast growth factor receptors are able to slow down functional decline and disease progression with an acceptable safety profile. While this is a major achievement, neither pirfenidone nor nintedanib cures IPF and most patients continue to experience disease progression and/or exacerbation despite treatment. Therefore, in recent years increasingly more attention has been paid to preservation of quality of life and, in the advanced phase of the disease, palliation of symptoms. Lung transplantation, the only curative treatment, remains a viable option for only a minority of highly selected patients. The unmet medical need in IPF remains high, and more efficacious and better tolerated drugs are urgently needed. However, a truly effective therapeutic approach should also address quality of life and highly prevalent concomitant conditions and complications of IPF.

## Introduction

The approach to treatment of idiopathic pulmonary fibrosis (IPF) has changed dramatically in the last decade. A number of factors have contributed to this, including improved, though still incomplete, knowledge of disease pathobiology, refined disease definition and diagnostic criteria, and advances in clinical trial design and conductance (1–3). Historically, corticosteroids and immunosuppressive agents have represented the standard of care for patients with IPF based on the prevailing hypothesis that chronic inflammation may precede and progresses to pulmonary fibrosis. However, the IPFnet-sponsored PANTHER-IPF (Evaluating the Effectiveness of Prednisone, Azathioprine, and N-acetylcysteine in Patients With IPF) trial was terminated prematurely following an interim safety analysis revealing that combination (triple) therapy of prednisone, azathioprine and N-acetylcysteine was associated with increased rates of all-cause mortality, hospitalization and serious adverse events compared to placebo (4). Accordingly, this therapy no longer represents a therapeutic option in patients with IPF (5).

Current paradigm of disease pathogenesis involves recurrent alveolar epithelial cell injury followed by an aberrant wound healing response characterized by uncontrolled migration and proliferation of lung fibroblasts and differentiation of fibroblasts to myofibroblasts resulting in excessive collagen deposition, scarring of the lung parenchyma and irreversible loss of function (6, 7). Accordingly, recent clinical trials have evaluated the efficacy of compounds targeting the wound healing cascade and fibrogenesis, but, overall, with disappointing results, probably because of the multitude of mediators, growth factors and signaling pathways involved in the fibrotic process (8). More recently, two compounds pleiotropic in their mechanisms of action—pirfenidone and nintedanib—have been approved for the treatment of IPF based on their ability to slow down the pace of functional decline and disease progression in phase 3 clinical trials (9, 10). Management of physical debility and palliation of symptoms are similarly important, while lung transplantation represents a realistic therapeutic option only in a small fraction of highly selected patients.

In this article, we summarize and discuss the most recent literature on pharmacological and non-pharmacological treatment of this dreadful disease.

## The ATS/ERS/JRS/ALAT guideline document on treatment of IPF

Originally published in 2011 (11), these evidence-based guidelines have been updated in 2015 to incorporate the most relevant data reported since publication of the previous document (5). For each treatment regimen, a multidisciplinary expert committee graded the certainty (e.g., the *confidence*) in effect estimate as *high, moderate, low*, or *very low* according to the GRADE (Grading of Recommendations Assessment, Development and Evaluation) methodology (12) and made a recommendation either “strong” or “conditional” *for* or *against* a given intervention. The recommendations were based, among others, on the strength of evidence, outcomes studies, and associated importance to patients, desirable and undesirable consequences of treatment, costs, feasibility of treatment, and acceptability of treatment to stakeholders. Current recommendations for treatment of IPF are summarized in Table 1.

**Table 1:** Key recommendations on pharmacological treatment of IPF according to current guideline.

	**2015 Guideline**	**2011 Guideline**
**THERAPEUTIC AGENT**		
Pirfenidone	Conditional recommendation for use^*^	Weak recommendation against use
Nintedanib	Conditional recommendation for use	Not addressed
Antiacid therapy	Conditional recommendation for use	Weak recommendation for use
Phosphodiesterase-5 inhibitor (sildenafil)	Conditional recommendation against use	Not addressed
Dual endothelin receptor antagonists (bosentan, macitentan)	Conditional recommendation against use	Strong recommendation against use
N-acetylcysteine (NAC)	Conditional recommendation against use	Weak recommendation against use
Azathioprine + corticosteroids + NAC	Strong recommendation against use	Weak recommendation against use
Warfarin	Strong recommendation against use	Weak recommendation against use
Imatinib	Strong recommendation against use	Not addressed
Selective endothelin receptor antagonist (ambrisentan)	Strong recommendation against use	Not addressed

* Conditional recommendations are synonymous with weak recommendations

Three therapeutic interventions received a *conditional* (e.g., weak) recommendation for use (e.g., pirfenidone, nintedanib, and antacid medication), and they are discussed below.

## Pirfenidone

Pirfenidone (5-methyl-1-phenyl-2-(1H)-pyridone) is an orally available, synthetic compound that exerts anti-fibrotic, anti-inflammatory, and anti-oxidant activities (13). While its exact mechanism of action remains to be elucidated, pirfenidone's biological effects are believed to occur mainly through suppression of tumor necrosis factor (TNF)-α, an early mediator of inflammation (14), and mediators in the transforming growth factor (TGF)-β pathway, such as the cytoplasmic Smad proteins (15), resulting in inhibition of fibroblast proliferation and differentiation to myofibroblasts, and decreased collagen production (16).

Four phase 3 randomized controlled trials have assessed the efficacy of pirfenidone in patients with IPF. In a Japanese study led by Taniguchi [Shionogi Phase 3 (SP3)], 275 patients were randomized in a 2:1:2 ratio to high-dose pirfenidone (1,800 mg/day), low-dose pirfenidone (1,200 mg/day), or placebo (17). As compared to placebo, both high-dose and low-dose pirfenidone reduced significantly the rate of decline in vital capacity (VC) (−0.16 vs. −0.09 L and −0.08 L; *p* = 0.042 and *p* = 0.039, respectively). Additional significant differences in favor of pirfenidone were observed in progression-free survival (PFS) (defined as decline in VC of >10% from baseline or death) and change in total lung capacity (TLC). Limitations of the study, however, included the change of the primary endpoint before unblinding and the handling of missing data (e.g., last observation carried forward, which may inflate the type 1 error rate). At the time of this trial, pirfenidone had already been approved for treatment of IPF in Japan based on a secondary endpoint analysis of a previous study [Shionogi Phase 2 (SP2)] showing a significantly reduced rate of acute exacerbations (AE) of IPF (AE-IPF) in patients randomized to pirfenidone (18).

The CAPACITY (Clinical Studies Assessing Pirfenidone in IPF: Research on Efficacy and Safety Outcomes) program consisted of two nearly identical trials (PIPF-004 and PIPF-006) that evaluated the efficacy of pirfenidone in IPF patients with mild to moderate functional impairment [predicted forced vital capacity (FVC) ≥50%, predicted carbon monoxide diffusing capacity (DL_CO_) ≥35%, either predicted FVC or predicted DL_CO_ ≤90%, and 6-minute walk test (6MWT) distance ≥150 m] (19). Study 004 enrolled 435 patients who were randomized in a 2:1:2 dosing ratio to pirfenidone 2,403 mg/day (*n* = 174), pirfenidone 1,197 mg/day (*n* = 87), or placebo (*n* = 174), whereas study 006 had only two arms (e.g., pirfenidone 2,403 mg/day, *n* = 173 and placebo, *n* = 171). The change in percentage predicted FVC from baseline to week 72 was the primary outcome in both trials. In study PIPF-004, mean FVC change at week 72 was −8.0% in the pirfenidone 2,403 mg/day arm and −12.4% in the placebo arm (*p* = 0.001). In addition, 35/174 (20%) patients in the pirfenidone 2,403 mg/day group vs. 60/174 (35%) in the placebo group had a decline in FVC of at least 10% (*p* = 0.001). In the pirfenidone low-dose group, change in FVC was intermediate to that of the pirfenidone 2,403 mg/day and placebo groups. Conversely, in study PIPF-006, the FVC change at week 72 did not differ significantly between the two groups (−9.0% in the pirfenidone group vs. −9.6% in the placebo group; *p* = 0.51). Based on these data, pirfenidone was approved by the European Medicines Agency (EMA), whereas the U.S. Food and Drug Administration (FDA) requested an additional phase 3 study to confirm drug efficacy before pirfenidone could be approved. The ASCEND (Assessment of Pirfenidone to Confirm Efficacy and Safety in IPF) trial enrolled 555 patients with IPF who were randomly assigned to either pirfenidone 2,403 mg/day (*n* = 278) or placebo (*n* = 277) (9). The primary outcome was the change in percentage of predicted FVC or death from baseline to week 52. Notably, in order to enrol patients at higher risk for disease progression, thus maximizing the likelihood of detecting a treatment effect, patients suspected to have airflow limitation [ratio of the forced expiratory volume in one second (FEV1) to FVC < 0.80] were excluded while the minimum DL_CO_ for enrolment was reduced from 35 to 30% of the predicted value. Pirfenidone treatment, as compared with placebo, was associated with a relative reduction of 47.9% in the proportion of patients who had an absolute decline of ≥10% in percentage predicted FVC or who died (46/278 [16.5%] vs. 88/277 [31.8%]; *p* < 0.001), and with a relative increase of 132.5% in the proportion of patients whose FVC remained stable (63/278 [22.7%] vs. 27/277 [9.7%]; *p* < 0.001). A series of sensitivity analyses corroborated the robustness of these findings and the magnitude of pirfenidone effect (20). Pirfenidone treatment was also associated with a reduced decline in the 6-minute walk distance (6MWD) (*p* = 0.04) and improved progression-free survival (defined as the time to a decrease of ≥10% in the percentage of the predicted FVC, a decrease of ≥50 m in the 6MWD, or death, whichever occurred first; *p* < 0.001). Conversely, pirfenidone was not superior to placebo with regard to dyspnea scores (*p* = 0.16), or all-cause (4.0 vs. 7.2%; *p* = 0.10) or IPF-related mortality (1.1 vs. 2.5%; *p* = 0.23). However, a pre-specified pooled analysis of the ASCEND and CAPACITY trials revealed that pirfenidone was associated with a significant reduction of both all-cause [3.5 vs. 6.7%; hazard ratio (HR): 0.52; *p* = 0.01] and IPF-related mortality (1.1 vs. 3.5%; HR: 0.32; *p* = 0.006) at week 52 compared with placebo (9). Pooled analyses of ASCEND and CAPACITY trials and meta-analyses, which included also data from the SP2 and SP3 Japanese trials, confirmed that pirfenidone treatment reduced significant the risk of mortality compared with placebo over 120 weeks (21). In addition, pooled analysis of the phase 3 clinical trials ASCEND and CAPACITY showed that pirfenidone beneficial effect extends to non-elective respiratory-related hospitalization, which is reduced by ~50% compared to placebo (7 vs. 12%, HR 0.52, *p*-value = 0.001) (22), and is consistent across a broad range of patient subsets (e.g., U.S. vs. non-U.S. patients, gender, age, race, various measures and degrees of lung function impairment, use of supplemental oxygen, smoking status, or time since diagnosis) (23, 24). Several recent publications, including “real world” experiences, have confirmed the long-term efficacy and safety profiles of pirfenidone in patients with IPF (25–27). These studies exemplified the difference between efficacy and effectiveness of pirfenidone use in patient with IPF, as pirfenidone was the first drug for IPF to show longitudinal effectiveness within the real-life clinical setting and not only within the “controlled” environment of a clinical trial (28).

Data on safety and efficacy of pirfenidone in patients with severe functional impairment (i.e., FVC% predicted <50% and/or DL_CO_ <35%) are limited. In a recent retrospective study of such patients (*n* = 43), pirfenidone was associated with a trend toward a reduced functional decline compared to the 6-month period preceding treatment initiation, but did not show any benefit after 1 year of treatment (29). At present, there are insufficient data to justify the use of pirfenidone in patients with severe functional impairment.

Common side effects of the drug include gastrointestinal intolerance (e.g., nausea, dyspepsia, vomiting, abdominal discomfort, diarrhea) and skin reactions (photosensitivity, rash), which in most cases are mild to moderate in severity, reversible, and without clinically significant sequelae. Gastrointestinal side effects can be prevented/mitigated by taking pirfenidone during a meal, following a gradual initial dosing titration, and taking prokinetic agents and/or proton-pump inhibitors, whereas avoiding direct sun exposure, applying a broad-spectrum sunscreen with high ultraviolet (UV) A and UVB protection, and wearing protective clothing generally reduces the risk of photosensitivity skin reactions (30). Rare cases of eosinophilic pneumonia have also been described (www.pneumotox.com). Pirfenidone has been granted approval for treatment of IPF by the FDA in October 2014.

## Nintedanib

Nintedanib, previously known by its development code BIBF 1120, is an intracellular inhibitor of the tyrosine kinases vascular endothelial growth factor receptor (VEGFR) 1-3, fibroblast growth factor receptor (FGFR) 1-3, and platelet-derived growth factor receptor (PDGFR) a and b (31). By inhibiting VEGFR, FGFR, and PDGFR, nintedanib interferes with a number of processes that have been implicated in the pathogenesis of IPF, namely proliferation and migration of primary human lung fibroblasts, fibroblast to myofibroblast transformation, and TGF-β-stimulated secretion and deposition of collagen by primary human lung fibroblasts, resulting in an inhibitory effect on extracellular matrix secretion and deposition (32). In patients with IPF, the safety and efficacy of four different doses of BIBF 1120 (e.g., 50 mg once daily [*n* = 86], and 50 mg [*n* = 86], 100 mg [*n* = 86] and 150 mg [*n* = 85] all twice daily) compared with placebo (*n* = 85) were initially evaluated in the TOMORROW (To Improve Pulmonary Fibrosis With BIBF 1120), a phase 2, proof-of-concept study, in which the primary endpoint was the annual rate of decline in FVC (33). In the BIBF 1120 150 mg twice daily group, FVC declined by 0.06 L per year compared with 0.19 L per year in the placebo group, corresponding to a 68.4% reduction in the rate of decline. In addition, BIBF 1120 150 mg twice daily was associated with a lower incidence of AE-IPF (15.7 vs. 2.4 vs. per 100-patient-years; risk ratio: 0.16; *p* = 0.02) and improved quality of life as assessed by the St. George's Respiratory Questionnaire (SGRQ) compared with placebo. The INPULSIS program consisted of two parallel 52-week, phase 3 trials (INPULSIS-1 and INPULSIS-2) designed to confirm the efficacy and safety of nintedanib 150 mg twice daily in patients with IPF (10). One thousand sixty-six patients were randomly assigned in a 3:2 ratio to either nintedanib 150 mg twice daily (*n* = 309 in INPULSIS-1 and *n* = 329 in INPULSIS-2) or placebo (*n* = 204 in INPULSIS-1 and *n* = 219 in INPULSIS-2). Similar to the TOMORROW trial, the primary outcome was the annual rate of decline in FVC. Both studies met the primary endpoint. Specifically, the adjusted annual rate of change in FVC was −114.7 ml in the nintedanib group and −239.9 ml in the placebo group in INPULSIS-1 (between group difference: 125.3 ml; *p* < 0.001) and −113.6 and −207.3 ml in INPULSIS-2 (between group difference: 93.7 ml; *p* < 0.001), respectively. In both trials, the robustness of the results of the primary analysis and the magnitude of treatment effect were confirmed by a series of prespecified sensitivity analyses. In addition, nintedanib treatment, compared with placebo, was associated with a reduced risk of disease progression—defined as absolute decline in percent predicted FVC of ≥10% or death—by 47% in INPULSIS-1 (24.3 vs. 40.7%; HR: 0.53; *p* = 0.0001) and by 33% in INPULSIS-2 (29.8 vs. 42.0%; HR: 0.67; *p* = 0.0054) (34). Furthermore, in both trials, patients receiving nintedanib were more likely to be stable (e.g., to have a decline in the percentage of predicted FVC of ≤5%) at week 52 compared with patients randomized to placebo (52.8 vs. 38.2% in INPULSIS-1, *p* = 0.001; and 53.2 vs. 39.3% in INPULSIS-2, *p* = 0.001) (32). A pooled analysis and a meta-analysis of data from the TOMORROW and INPULSIS trials confirmed the beneficial effect of nintedanib in slowing down disease progression (35).

Time to first investigator-reported AE, one of the two key secondary end points (the other being SGRQ), was significantly delayed with nintedanib vs. placebo in INPULSIS-2 (HR: 0.38, *p* = 0.005) but not in INPULSIS-1 (HR: 1.15, *p* = 0.67). However, a pre-specified sensitivity analysis of pooled data from the INPULSIS trials showed that nintedanib compared to placebo delayed significantly the first adjudicated AE-IPF (either confirmed or suspected) (HR: 0.32, *p* = 0.001) (10). More extensive analysis of the INPULSIS data showed that nintedanib treatment reduces by ~40% mortality following AE, although this result did not reach statistical significance (36). Nintedanib treatment was associated with a significantly smaller increase in the total SGRQ score (consistent with more preserved quality of life) in INPULSIS-2 (2.80 points vs. 5.48 points in the placebo group; *p* = 0.02) but not in INPULSIS-1 (4.34 points vs. 4.39 points, respectively; *p* = 0.97). In addition, in a pre-specified analysis of pooled data from the INPULSIS trials, the adjusted mean change in the SGRQ total score from baseline to week 52 was similar in the nintedanib and placebo groups. Finally, in a pre-specified pooled analysis of the INPULSIS data, the nintedanib and placebo arms did not differ significantly in terms of death from any cause (5.5 vs. 7.8%, respectively; HR: 0.70; *p* = 0.14) or death from a respiratory cause (3.8 vs. 5.0%, respectively; HR: 0.74; *p* = 0.34).

IPF is a highly heterogeneous disease and patients with varying clinical phenotypes may respond differently to antifibrotic therapies. A number of subgroup analyses however have confirmed the broad therapeutic efficacy of nintedanib in patients with IPF. Using pooled data from the INPULSIS trials, Costabel and colleagues showed that treatment effects, examined against sex, age (<65, ≥65 years), race (White, Asian), smoking status (never, ex/current), baseline FVC % predicted (≤70%, >70%), baseline SGRQ total score (≤40, >40), corticosteroid use (yes, no) and bronchodilator use (yes, no) did not differ significantly for the primary (annual rate of decline in FVC) or key secondary (time to first AE and change from baseline in the SGRQ) end points (37). In a *post-hoc* subgroup analysis of pooled data from the INPULSIS trials (*n* = 1,061), Raghu and colleagues demonstrated that the rate of decline in FVC in patients with possible UIP on high-resolution CT (HRCT) [i.e., reticular abnormality and traction bronchiectasis in the absence of features inconsistent with usual interstitial pneumonia (UIP)] and no confirmatory surgical lung biopsy is similar as in patients with a diagnosis of IPF according to current guidelines (i.e., honeycombing on HRCT and/or UIP on surgical lung biopsy) (38). A further *post-hoc* subgroup analysis of pooled data from the INPULSIS trials revealed that patients with IPF and preserved lung volumes (FVC >90% predicted) experience the same rate of functional decline and receive the same benefit from nintedanib as patients with more impaired lung function (FVC <90%), thus supporting the concept of offering early treatment to patients with IPF (39).

The most frequent adverse event associated with nintedanib treatment was diarrhea (~60% within the first 3 months of treatment), which in most cases was of mild or moderate intensity and led to premature study discontinuation in 4.5% of patients (vs. none in the placebo group) in INPULSIS-1 and 4.3% of patients (vs. 0.5% in the placebo group) in INPULSIS-2 (10). However, in both trials, the same proportion of patients in the nintedanib and placebo groups experienced serious adverse events. Nintedanib has been approved by the FDA in October 2014 and in Europe in early 2015.

## Antacid therapy

Gastroesophageal reflux (GER), both symptomatic and asymptomatic, occurs in a high proportion of patients with IPF, and chronic microaspiration secondary to GER is believed to play a role in the pathogenesis and progression of the disease (40, 41). Accordingly, a number of studies have explored the possibility that antacid therapy (AAT) may be beneficial in terms of slowing disease progression and even improving survival in patients with IPF. In an uncontrolled retrospective study of 204 IPF patients from two major academic medical centers in the U.S., GER medications [either proton pump inhibitors (PPI) or H2 blockers] were associated with reduced radiological fibrosis and improved survival (42). Furthermore, a *post-hoc* analysis of data from patients randomized to placebo in three IPFnet-sponsored clinical trials (*n* = 242, 124 of whom [51%] were taking either PPI or H2 blockers at the time of enrolment) showed that the use of AAT was associated with a smaller decrease in FVC (estimated change over 30-weeks of −0.06 vs. −0.12 L in patients not taking AAT; *p* = 0.05) and fewer AEs (no events vs. 9 events in patients not taking AAT; *p* < 0.01) (43). The 2015 guidelines conditionally recommend the use of GER medications in patients with IPF based on the potential benefit and favorable side effect profile of these drugs (5). However, a more recent *post-hoc* analysis of patients assigned to placebo in three clinical trials of pirfenidone (CAPACITY 004, CAPACITY 006, and ASCEND) (*n* = 624, 291 of whom [47%] received AAT) questioned the efficacy of GER medications in IPF (44). Indeed, in this study AAT did not improve progression-free survival (defined as FVC decrease ≥10%, 6MWD decrease ≥50 m, or death), FVC decline, hospitalization and all-cause and IPF-related mortality. Moreover, use of GER medications was associated with a significantly higher rate of overall infections (*p* = 0.02) and pulmonary infections (*p* = 0.02) in patients with advanced IPF (e.g., FVC <70%). The role of AAT in the treatment of patients with IPF remains highly controversial and needs to be addressed in prospective randomized trials (45). One such study, which is currently ongoing, will test the hypothesis that treatment with laparoscopic antireflux surgery in patients with IPF and abnormal GER (WRAP-IPF; NCT01982968) may slow the decline in FVC over the 48-week study duration by abolishing acid and non-acid reflux, both believed to be pathogenic in IPF (41).

## Management of acute exacerbations

The term “acute exacerbations” (AE) refers to episodes of acute respiratory deterioration accompanied by the development of new radiologic abnormalities (i.e., ground glass opacities and/or consolidation on a background of reticulation and traction bronchiectasis with or without honeycomb changes) on chest X-ray or HRCT (46). The annual incidence of AE ranges between 4 and 20% and is significantly higher in patients with more severe disease (46). The prognosis following an AE is poor with a median survival of ~3 months (47). At present, there are no therapies of proven efficacy for AE-IPF so that the treating physician is left with supportive care (i.e., palliation of symptoms and relief of hypoxemia with supplemental oxygen) and unproven interventions. Therefore, searching for (and ruling out) known causes of clinical deterioration, including drug toxicity, is warranted.

### Corticosteroids

The 2011 guidelines make a weak recommendation *for* the use of corticosteroids in patients with AE-IPF, although randomized controlled clinical trials are lacking (11). The appropriate dose and duration of therapy remain unclear, but in most series the dose has ranged between prednisone 1 mg/kg per day orally and methylprednisolone 1 g per day intravenously for 3 days followed by a gradual taper, based on clinical response. The role of high-dose corticosteroids in AE-IPF remains highly controversial (48–50).

### Antibiotics

The rationale behind the use of broad-spectrum antibiotics to treat AE-IPF is that many patients present with flu-like symptoms and have elevated neutrophil count in bronchoalveolar lavage fluid (51). More recently, in a retrospective single-center study of IPF patients hospitalized for AE (*n* = 85), Kawamura and co-workers showed that early administration of azithromycin 500 mg/day for 5 days is associated with a significantly lower mortality compared with a fluoroquinolone-based regimen (26 vs. 70%; *p* < 0.001) (52). This study however has a number of limitations, including its small sample size, retrospective nature and the choice of fluoroquinolone-treated patients as control group; accordingly, these findings need to be confirmed in prospective studies. Ding and colleagues evaluated the use of procalcitonin (PCT)-guided antibiotic treatment vs. standard clinician-determined antibiotic treatment in patients with AE-IPF (53). PCT guidance reduced significantly the duration of antibiotic use, but the duration of mechanical ventilation and overall mortality were similar in both groups.

### Mechanical ventilation

The role of invasive mechanical ventilation (IMV) and non-invasive ventilation (NIV) in the management of AE-IPF has not been formally studied and remains unclear. The 2011 guideline document makes a weak recommendation *against* the use of MV to treat AE-IPF, thus suggesting this may be a reasonable intervention only in a minority of selected patients (11); however, a recent U.S. nationwide retrospective cohort analysis suggested that mortality rates of IPF patients who received IMV or NIV for acute respiratory failure (51.6 and 30.9%, respectively, in this study) may be lower than previously reported (54). Prospective studies are needed to identify IPF patients more likely to benefit from MV and NIV.

### Novel approaches

#### Recombinant human soluble thrombomodulin (rhTM)

It is a regulator of intravascular coagulation expressed on the endothelial cell surface (55). A number of studies have consistently shown that rhTM improves 3-month survival in patients with AE-IPF (56–58). However, these results need to be confirmed in the setting of randomized controlled trials.

#### Hemoperfusion with polymyxin B immobilized fiber

Originally developed to remove gram-negative bacterial endotoxins, polymyxin B direct hemoperfusion (PMX-DHP) may also remove cytokines involved in lung injury (59). A number of retrospective studies, mostly from Japan, have shown that PMX-DHP improves oxygenation and survival in patients with AE-IPF, although most patients received also high-dose systemic corticosteroids (60–62). PMX-DHP is a promising therapeutic approach in patients with AE-IPF, but its safety and efficacy need to be validated in larger prospective clinical trials.

#### Autoantibody-targeted treatment

Recent evidence suggests that immune dysregulation may contribute to IPF progression and that treatments that reduce autoantibodies may be beneficial in a significant minority of patients (63). Donahoe and colleagues treated 11 patients with AE-IPF with plasma exchange and rituximab ± intravenous immunoglobulin (64). Compared to historical controls treated with high-dose corticosteroids, trial subjects had significantly better 1-year survival (1/20, 5% vs. 9/11, 82%). These data suggest considering a trial of autoantibody-targeted therapies in patients with AE-IPF.

## Mesenchymal stem cells

Mesenchymal stem cells (MSCs) are multipotent stromal cells with the potential of transdifferentiation, clonality, and self-renewal. MSC properties include also immunomodulation, epithelial repair, and secretion of growth factors (65). MSCs have been shown to ameliorate inflammation and mitigate parenchymal remodeling in bleomycin-induced pulmonary fibrosis (66), but the bleomycin model recapitulates only partially the complex pathobiology of IPF (67). Therefore, the application of MSCs in patients with IPF is controversial and under study (68). In a small cohort of patients with IPF (*n* = 14), Tzouvelekis and colleagues have shown that endobronchial infusion of autologous adipose derived stem cells was not associated with serious adverse events (69). Yet, results should be interpreted cautiously before rigid conclusions can be drawn. Significant limitations severely hampering the widespread implementation of stem cell use in IPF relate mainly to our limited knowledge of the fate of these cells within the pro-fibrotic microenvironment given their mesenchymal origin and their potential to differentiate into myofibroblasts, thus causing disease progression. In addition, there are many unanswered questions including the time (early or advanced disease) and optimal route of administration (intravenous or endobronchial), source of mesenchymal stem cells (MSCs) (eg, adipose tissue, bone marrow, or umbilical cord), frequency of infusions as well as the choice of the appropriate primary end-points to show benefit (70). An FDA approved RCT investigating safety and efficacy of a single intravenous administration of allogeneic bone-marrow derived MSCs is currently recruiting patients (NCT02611167) and results are eagerly awaited.

## Non-pharmacological management

**Figure 1:**
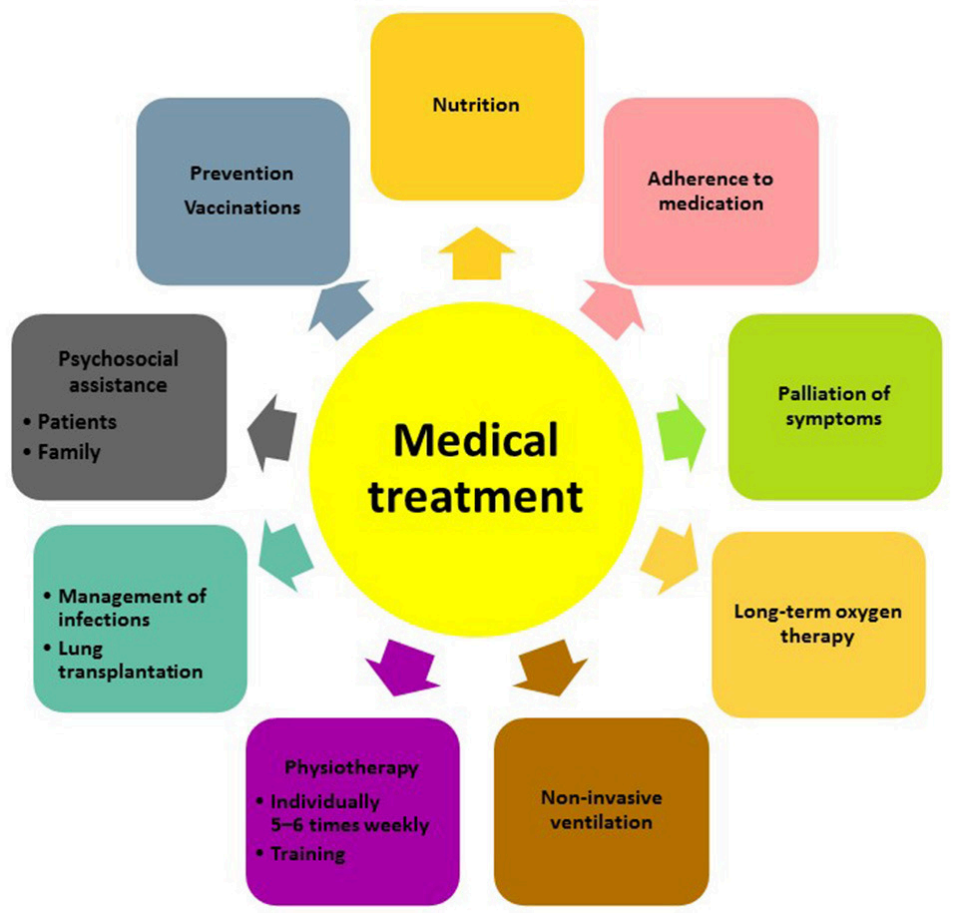
Multidisciplinary approach to the management of patients with IPF.

Besides pharmacological treatment and lung transplantation, there is increasing evidence that supportive measures such as pulmonary rehabilitation, adequate nutrition, prevention of infections and timely initiation of palliative care can improve and maintain health status and quality of life of patients with IPF (Figure 1). The most recent advances in lung transplantation, rehabilitation, and palliative care are discussed below.

### Lung transplantation

In the last 5 years, IPF has become the most common indication for lung transplantation (71). In the 2011 guideline document, lung transplantation was strongly recommended in IPF, though in highly selected patients (11). According to the Organ Procurement and Transplantation Network (OPTN) report, the proportion of patients transplanted with IPF has increased constantly in recent years and reached 49.6% in 2015. At present, the worldwide frequency of the procedure is ~4,000 per year with 5-year survival rates ranging from 50 to 60%, whereas 10 years survival is around 30% (72). Recipients aged 65 years or older and those with a *lung allocation score* (LAS) of 60 or higher show the lowest survival (72). Recent analyses performed after implementation of the LAS suggest that lung transplantation in patients older than 70 years of age may have outcomes comparable to those of younger patients (73). The trend in transplanting older patients may increase in the future due to greater experience of transplant centers and the raising awareness of IPF patient advocacy groups, which in the IPF Charter on patients' rights raised the point that a*ge restrictions for lung transplantations exclude many healthy, viable patients* (74).

Worldwide, bilateral lung transplants are preferentially performed (about 70% of all procedures) (72), while most patients with interstitial lung disease (ILD) undergo single lung transplant (75). Bilateral lung transplants in patients with IPF appear to be associated with longer survival, although the long-term survival advantage is counterbalanced by longer time on the waiting list and higher risk of mortality (76). There are several factors that influence the selection procedure, such as age, comorbidities, anatomical features, predicted pre-transplant survival, organ availability, and center experience, but further long-term data are needed to draw firm conclusions on this highly debated topic (77). Early referral of IPF patients to transplant is highly recommended due to their poor prognosis, high mortality on the waiting list and the unpredictable disease course. After the LAS implementation and the revision of the selection criteria (78), change over time in lung function (FVC and DL_CO_) has become the driving factor for early referral to lung transplant and has led to a reduction of the waiting list mortality of 10–20% (72).

Overall, the median survival of patients with ILD after lung transplant is 4.7 years, significantly less than that of patients with chronic obstructive pulmonary disease (COPD) and cystic fibrosis (CF) (5.5 and 8.3 years, respectively) (71, 72). Older age at the time of the transplant, prevalence of age-related comorbidities, and higher prevalence of bronchiolitis obliterans syndrome (BOS) represent the main factors that influence outcome (77). Although the incidence of pulmonary and extra-pulmonary post-transplant complications does not appear to be higher in patients with IPF than in those with other ILDs or lung diseases, there is an increasing risk for IPF patients to develop thromboembolism before and after lung transplantation (79). In addition, GER, which is highly prevalent in patients with IPF (80) and often complicates lung transplantation, is strongly associated with the development of BOS (81) and its presence should be carefully evaluated both pre- and post-transplantation (77). Fundoplication after lung transplantation and antireflux surgery pre- and post-transplantation have shown to preserve lung function and prevent reflux-associated BOS (82). On the other hand, there are no data to suggest that IPF patients are at higher risk to develop restrictive chronic lung allograft dysfunction (CLAD), a condition characterized by concomitant decrease in FVC and FEV_1_, and usually accompanied by parenchymal infiltrates (77).

### Pulmonary rehabilitation

The natural history of IPF is characterized by a progressive impairment of exercise capacity and mobility, as a consequence of shortness of breath and exercise-induced hypoxemia, which induce patients to reduce and eventually avoid physical activity (83). In addition, sarcopenia, the age-related loss of muscle mass quality and strength, contributes to inactivity in these patients, which are typically 60–80 years old. In the general population and in chronic respiratory diseases, inactivity is associated with poorer health-related outcomes, including higher mortality risk (84). Exercise training in healthy subjects has been shown to positively affect the physiology of cardiovascular, respiratory, and musculoskeletal systems (85). In ILDs, pulmonary rehabilitation has been shown to alleviate respiratory and psychological symptoms, particularly dyspnea and anxiety, and improve exercise tolerance, 6MWD and quality of life scores (86–88). Possible mechanisms underlying these beneficial effects include chest expansion during deep-breathing exercises and stretching of the thoracic muscles resulting in a more efficient breathing pattern, improved respiratory muscle strength, enhanced pleural elasticity and pulmonary compliance (89).

The majority of studies on pulmonary rehabilitation in IPF combined aerobic activity (walking and/or cycling) with resistance and flexibility exercises for peripheral skeletal muscles (90). In a recent meta-analysis, 9 out of 10 of the exercise training studies examined showed a benefit in 6MWD (range 35–81 meters), peak aerobic capacity, and improvement of dyspnea and quality of life (91). Overall, supervised exercise training programs appear to provide the best results in terms of compliance and maintenance of physical activity, while home-based programs seem to be associated with a lower level of improvement (92). Pulmonary rehabilitation should be considered at any stage of IPF and in patients awaiting lung transplantation, since its timely administration correlates with a clinically significant improvement in physical activity and health-related quality of life (93). While the short-term effects of pulmonary rehabilitation on IPF outcomes are supported by several retrospective and prospective studies, its long-term effects have not been extensively studied and systematic investigation in this regard is needed.

### Oxygen treatment

Prescription of long-term oxygen treatment (LTOT) is a challenging step in the management of IPF not only for the patients but also for their relatives and treating physicians. Patients tend not to accept LTOT as the disease becomes more “visible.” In addition, their training with the devices is often inadequate and problematic. Although supplemental oxygen therapy is likely to improve symptoms and overall quality of life in IPF patients, especially those with resting or nocturnal hypoxemia (11), a recent systematic review showed no effects of oxygen therapy on dyspnoea during exercise in ILD, although exercise capacity was increased (94). The *ambulatory oxygen in fibrotic lung disease* (AmbOx) trial is the first randomized control trial investigating the effects of ambulatory oxygen during daily life on health status and breathlessness in patients with ILD (95). In this study, patients with fibrotic lung disease with oxygen saturation (SaO_2_) ≥94% at rest but ≤88% during a 6MWT were treated with ambulatory oxygen for a 2-week period compared to 2 weeks off. Preliminary data show that ambulatory oxygen is associated with significantly improved health status in patients with ILD (96).

### Palliation of symptoms

The aim of palliative care in IPF is to reduce the impact of symptoms on quality of life and minimize stress and psychological consequences, mainly depression and anxiety, which are related to the inexorably progressive nature of the disease. Dyspnea and cough appear early in the disease course, while fatigue, reduced appetite, and weight loss are typically seen in advanced stages of the disease. Dyspnea is strongly associated with reduced quality of life (97, 98) and has been shown to correlate with a worse prognosis (99). The mechanisms behind dyspnea are not fully understood but neuroimaging studies have shown how dyspnea and pain activate common areas in the brain and share a cerebral network (100). In a recent systematic review on the use of opioids for dyspnea in patients with IPF, Kohberg and colleagues observed that only systemic morphine administration improved significantly the dyspnea score on a visual analog scale without severe side effects (101). The majority of these studies used an individually titrated dose between 10 and 30 mg, which appears to be associated with a beneficial effect on dyspnea. Conversely, nebulized morphine did not show any effect on dyspnea, although this was probably due to the sub-therapeutic dosage (102). The concern of morphine-induced respiratory depression was addressed by almost all studies, but only minor side effects, such as nausea and constipation, were reported (103). However, strict dosage is necessary, and the risk of tolerance should be considered. Only randomized placebo-controlled trials will clarify whether morphine is effective and safe in the treatment of dyspnea in patients with IPF.

Chronic cough is another major issue in the management of IPF, since patients are often refractory to conventional anti-tussive therapy (104). A small non-randomized study with oral corticosteroids showed a reduction of cough reflex in IPF patients (103), somehow supporting the beneficial effect of low-dose steroids observed in clinical practice (102). Thalidomide has also been investigated as a potential treatment of cough in a single-center study, but despite the positive effect on quality of life, only 20% of the subjects completed the study due to adverse events (104). Recently, Birring and colleagues assessed the safety and efficacy of PA101, a novel formulation of sodium cromoglicate delivered via a high-efficiency eFlow nebuliser, in patients with IPF and chronic cough (105). IPF patients and patients with chronic idiopathic cough (CIC) were randomized 1:1 to receive PA101 (40 mg) or placebo three times daily for 2 weeks, followed by a 2-week washout, and then crossed over to the other arm. Compared to placebo, PA101 reduced daytime cough frequency by 31% at day 14 in patients with IPF but not in those with CIC, suggesting that the mechanism of cough in IPF may be disease specific. More recently, pirfenidone has been shown to significantly reduce objective 24-h cough counts and to improve subjective measures of cough, although the study had a short follow-up period and was not placebo-controlled (106).

Access to palliative care is one of the most relevant unmet needs in the management of IPF (74). It is, therefore, strongly recommended to treat respiratory symptoms irrespective of disease severity (11). Early referral of patients to individual counseling, patient support groups and comprehensive rehabilitation programs, which should also include psychological support, can have a positive impact on perception of dyspnea and quality of life, and prepare patients to face the final stages of the disease (107, 108).

### Outlook

With improved clinical and basic understanding of IPF, an evidence-based approach to treatment is evolving. However, access to approved therapies remains suboptimal and problematic. Maher and colleagues have recently reported that ~40% of patients with confirmed IPF across Europe do not receive antifibrotic treatment (109). In particular, there appears to be a tendency to adopt a “wait and watch” approach in patients with mild functional impairment or stable disease. This observation underscores the need for increasing physician awareness of the progressive nature of IPF and the benefit associated with early treatment. In addition, compared to treated patients, a lower proportion of untreated patients had a multidisciplinary team evaluation at diagnosis, highlighting the importance of encouraging and facilitating patient referral to expert centers.

The need for safer and more efficacious treatment options has led to an exponential increase in the number of high-quality clinical trials of pharmacological interventions (Table 2). Yet, drug development in IPF poses major challenges, ranging from the lack of animal models that mimic all pathologic changes of IPF to the choice of the appropriate primary outcome on which to judge drug efficacy and the clinical meaningfulness of the observed effects. FVC is acknowledged as the preferred endpoint in IPF clinical trials (110), but how often the FVC should be measured remains unknown. Indeed, while the preferred time interval has been every 3 months, this may result in “missing events” in the case of patients who progress and die within this timeframe without documentation of a significant decline of their FVC (111). Recently, Russell and colleagues have shown that unsupervised daily home spirometry is a feasible and clinically informative tool for monitoring disease behavior in patients with IPF (112). From a clinical trial perspective, daily home spirometry may significantly reduce both the required sample size and duration of the trial by increasing the number of recorded measurements. Daily home spirometry may benefit particularly early phase clinical trials.

**Table 2:** Most developed drug candidates for idiopathic pulmonary fibrosis.

**Compound**	**Mechanism of action**	**Study design**	**Primary outcome/study duration**	**Developmental phase/status**	**Clinical trial identifier**
Inhaled TD139	Galectin-3 inhibitor	Phase 1: randomized, placebo-controlled, single ascending dose. Phase 2: randomized, placebo-controlled, multiple dose expansion cohort	Safety and tolerability (number of participant with adverse events over 2 weeks)	Phase I/II; completed, awaiting results	NCT02257177
PRM-151	Recombinant human Pentraxin-2 (serum amyloid P). Antifibrotic immunomodulator.	Randomized, placebo-controlled	Change in FVC % predicted from baseline through week 28	Phase II; active, not recruiting	NCT02550873
KD025	ROCK2 inhibitor	Randomized, open-label, active comparator	Change in FVC from baseline through week 24	Phase II; recruiting	NCT02688647
Tipelukast	LT receptor antagonist, PDE 3 and 4 inhibitor, 5-LO inhibitor	Randomized, placebo-controlled	Change in FVC from baseline through week 26	Phase II; recruiting	NCT02503657
PBI-4050	CTGF, α-SMA and collagen I expression inhibitor	Open-label, single-arm	Safety and tolerability (number of participant with abnormal laboratory values and/or adverse events over 9 months)	Phase II; completed, awaiting results	NCT02538536
GLPG1690	Autotaxin inhibitor	Randomized, placebo-controlled	Safety and tolerability over 12 weeks; pharmacokinetics; concentration of lysophosphatidic acid in blood/bronchoalveolar lavage	Phase II; Completed, awaiting results	NCT02738801
CC-90001	JNK inhibitor	Randomized, placebo-controlled	Change in FVC % predicted from baseline through week 24	Phase II; recruiting	NCT03142191
BMS-986020	LPA receptor inhibitor	Randomized, placebo-controlled	Rate of change in FVC at week 26	Phase II; completed, awaiting results	NCT01766817
BG00011 (formerly STX-100)	α_v_β_6_ inhibitor	Randomized, placebo-controlled, dose escalation	Safety and tolerability (number of participant experiencing adverse events over 16 weeks)	Phase II; completed, awaiting results	NCT01371305
Pamrevlumab/FG-3019	CTGF inhibitor	Randomized, placebo-controlled	Change in FVC from baseline through week 48	Phase II; active, not recruiting	NCT01890265
Rituximab	CD20 inhibitor	Randomized, placebo-controlled	Change in titers of Autoantibodies to HEp-2 Cells over 9 months	Phase II; completed, awaiting results	NCT01969409
Lebrikizumab	IL-13 inhibitor	Randomized, placebo-controlled and active drug (i.e., pirfenidone) controlled	Rate of decline in FVC % predicted from baseline through week 52	Phase II; completed, awaiting results	NCT01872689
SAR156597	IL-4 and IL-13 inhibitor	Randomized, placebo-controlled	Absolute change in FVC from baseline through week 52	Phase II; completed, awaiting results	NCT02345070

*CTGF, connective tissue growth factor; FVC, forced vital capacity; HEp-2, Human epithelial type 2; IL, interleukin; JNK, c-Jun N-Terminal Kinase; 5-LO, 5-lipoxygenase; LPA, lysophosphatidic acid; LT, leukotriene; PDE, phosphodiesterase; ROCK2, Rho associated kinase 2; α-SMA, α-smooth muscle actin; TGF-β, transforming growth factor-β*.

## Conclusions

The management of patients with IPF is highly complex due to the progressive nature of the disease, the debilitating symptoms that severely impair quality of life and the highly prevalent comorbidities and complications. Currently, there is significant lack of knowledge regarding management of comorbid conditions including pulmonary hypertension and lung cancer as well as disease acute exacerbations that severely limit patients' survival. In the terminal phase of the disease palliative care becomes critically important. Developing a real cure for patients suffering from this terrible disease requires a close collaborative interplay between the scientific, professional, and patient community and the pharmaceutical industry. Only a comprehensive approach to disease management will eventually prove truly efficacious. Clinical trials focusing on disease biology and mechanisms by applying personalized medicine approaches seems to be the way forward (113, 114).

## Author contributions

PS: conception and drafting of the article, final approval of the manuscript; AT and FB: drafting and critical revision of the article, final approval of the manuscript.

### Conflict of interest statement

PS has served as consultant for InterMune, Roche, Zambon, PPM Services, and Santhera Pharmaceuticals, has served on scientific advisory boards for Boehringer Ingelheim and Galapagos, has been a lecturer at symposia organized by InterMune, Roche/Genentech, Boehringer Ingelheim, and Novartis, and has received travel grants from InterMune, Roche, Boehringer, and Zambon. FB has received speaker fees, advisory board honoraria or travel grants from InterMune, Boehringer Ingelheim, Serendex, and Roche. AT is inventor of a therapeutic patent entitled “Inhaled or aerosolized delivery of thyroid hormone to the lung as a novel therapeutic agent in fibrotic lung diseases” OCR#6368 (the “Invention”), disclosed to Yale University.
